# Does Happiness Launch More Businesses? Affect, Gender, and Entrepreneurial Intention

**DOI:** 10.3390/ijerph17186908

**Published:** 2020-09-21

**Authors:** Gloria Sweida, Cynthia L. Sherman

**Affiliations:** 1 School of Business, Southern Illinois University Edwardsville, Edwardsville, IL 62026, USA; 2 Martin V. Smith School of Business & Economics, California State University Channel Islands, Camarillo, CA 93012, USA; cynthia.sherman@csuci.edu

**Keywords:** positive affect, negative affect, entrepreneurial intention, gender, gender roles

## Abstract

In one of the first studies to examine how positive affect, negative affect, gender, and gender roles interact with entrepreneurial intention, we conducted an online survey of 849 adults from the western, midwestern, and southern regions of the United States. A higher positive affect was associated with greater intention to start a business, however, lower levels of negative affect were not. As in previous studies, women showed less entrepreneurial intention than men, however, the presence of positive affect had a larger positive impact on women’s entrepreneurial intention than men’s. Contrary to expectations, acceptance of traditional gender roles interacted with entrepreneurial intention such that women’s entrepreneurial intention increased as their support of traditional gender roles increased, and for men, entrepreneurial intention decreased slightly as acceptance of traditional gender roles increased.

## 1. Introduction

As we explore the myriad of ways that affect, emotion, and mood drive human behavior, particularly in the workplace [1,2], we also find ways by which they drive entrepreneurial behavior. Many research teams have investigated the role played by the broad construct of affect in entrepreneurship. While many studies conflate the states of emotion and mood with trait-level affect, some researchers have argued that an individual’s frequent experience of positive emotions and positive moods is not meaningfully distinguishable from dispositional or trait-level affect [2,3,4]. In their study, Watson et al. [4] asked respondents to estimate their mood states across both short and long time frames, and because of the strong relationship between trait-level affect and emotional experiences based on shorter time frames, we feel confident in drawing upon the literature that includes measures of emotion, mood and affect, which we will include under the general term “affect”.

Baron [5] considered the role of affect in decision-making, opportunity identification, and coping with adversity, all of which are important aspects of the entrepreneurial process. Cardon et al. [6] considered positive affect a key component of entrepreneurial passion, which is connected to persistence and creative problem solving, and therefore positive outcomes for nascent ventures. Further, other research teams have found that entrepreneurs report high work and life satisfaction [7,8,9]. Affect may also play a role in successive entrepreneurial endeavors because of the connection to entrepreneurial passion and persistence [6,10]. Stenholm and Nielsen’s [10] study looked at how entrepreneurial passion was created from emotional support. They found that emotional support helped to create positive emotions that helped entrepreneurs to engage with their environment, changing their perceptions of the environment, ideas, and persons more favorably, as described by Fredrickson’s broaden and build theory [11].

Hayward and colleagues [12] theorized that the upward spirals of positive affect described in broaden and build theory [11] can be a useful framework for viewing success in serial venture creation. This is because the positive affect experienced in earlier ventures builds relationships and self-efficacy, providing resources that entrepreneurs can call upon when building successive ventures. However, there can be no venture creation or positive economic and social outcomes due to venture creation without a would-be entrepreneur expressing the desire and plan to start a business.

However, little is known about how positive and negative affect, directly and indirectly, impacts entrepreneurial intention (EINT). In one of the few inquiries directly investigating the role of affect on EINT, Grichnik and colleagues [13] examined the role of emotional states on the cognitive evaluation of business opportunities and one’s motivation to act on that opportunity. They found that positive emotional states make one significantly more likely to evaluate a business opportunity positively, yet less likely to act upon that opportunity. Further, the presence of strong emotions, both negative and positive, make one less likely to decide to dedicate more resources to an opportunity, regardless of how it is evaluated. Grichnik and colleagues [13] note that the affect in entrepreneurship may play an even more important role due to the uncertainty of the environment and that the decision-making tasks are impacted by affect.

Furthermore, there is reason to believe that affect interacts with gender in ways that influence EINT. Based on Global Entrepreneurship Monitor 2018/2019 Women’s Entrepreneurship Report, [14] reported that women’s total entrepreneurial activity (TEA, i.e., nascent or new entrepreneurs of adult working age) was three quarters that of men’s TEA. Women’s TEA is approximately 10% of the global population whereas men’s TEA is 13%. Accordingly, men tend to express higher EINT than women [15,16]. This gap in TEA and EINT, though narrowing, indicates that women do not take advantage of the opportunity to access the emotional and financial benefits that come with entrepreneurship as their male counterparts.

Revealing ways that positive and negative affect impact the desire and plan to start a business and recognizing the role of gender and gender roles will help would-be entrepreneurs access the internal resources necessary to start a business. In the present study, we examine how positive and negative affect interact with gender and acceptance of gender roles to impact EINT. We provide a review of the literature on the influence of affect on entrepreneurship and EINT, and the role of gender and acceptance of traditional gender roles in entrepreneurship. We report the results from a survey of 849 adults and discuss the implications of the findings.

### 1.1. Affect, Emotion, and Mood

Human behavior is guided by emotion, mood, and more broadly by affect [1,2]. Affect, emotion, and mood are often conflated; however, affect refers to a more general state of consciously accessible feelings [2,17]. In contrast, emotions are defined as a response to a stimulus, unfolding over a relatively short time [2,11]. Moods are defined as somewhat stable background sensations that are not associated with a particular stimulus [2,17]. Affect, more generally, can be considered the accumulation of the experience of positive or negative moods and emotions. The creators of the widely used Positive and Negative Affect Scale (PANAS), Watson and colleagues [4], created the PANAS as a two-factor construct, therefore, one can have positive and negative emotions at the same time. They also determined that emotions and moods are indeed generally reflective of one’s dispositional-level affect. As such, even though both positive and negative affect can be conceived as both a state and trait, in this study, we study trait/dispositional-level affect. [2,3].

Affect is a form of information that influences cognitive processes such as perception, judgment, decision, memory, creativity, and coping with stress [5]. Schwarz and Clore’s [18] affect as information theory suggests that positive or negative affect act as categories for organizing experiences and making similar material easier to retrieve. Several researchers have utilized this theory [5,19,20] to posit that affect impacts cognition by priming memories and associations, and by serving as a heuristic for classifying and responding to objects, ideas, and people.

### 1.2. Positive Affect

Positive affect includes emotions such as joy, hope, and inspiration [21]. Positive affect facilitates approach behavior and prompts people to engage with their environment. Engaging with one’s environment tends to facilitate the acquisition of resources in the external world [11]. The resources accrued during states of positive emotions outlast the transient emotional states that led to their acquisition. The positive emotions can lead to the urge to explore the environment, take in new information, and expand the self [22,23]. From an entrepreneurial perspective, for instance, this could lead to creative ideation or a playful investigation that might lead to a new product or business. Therefore, the often-incidental effect of experiencing a positive emotion has the impact of leading to an increase in one’s resources [11] which can positively impact one’s overall personal, psychological, professional, and physical well-being.

Positive affect also helps build social capital and psychological well-being, which leads to personal growth, meaningful goals, and self-acceptance [24]. Additionally, those with more positive affect experience more positive outcomes in work, health, and relationships [11,25,26]. This may be because positive affect serves as a conduit for the integration of new ideas into current knowledge. The new knowledge can inspire innovative and creative thinking about goal attainment.

Positive affect broadens thinking and enhances resilience and the ability to cope with challenging situations [11,27]. Positive emotions, over time, may loosen the hold that negative emotion has on one’s reactions to events, such as the flee response to something fearful. Positive affect can broaden a person’s momentary thought–action repertoire to include ideas of play or exploration. Positive affect also yields better health outcomes, as the frequent experience of positive emotions yields a faster recovery time from cardiovascular stress, lessening damage to the body over time [11].

Positive affect influences several key entrepreneurial processes. Trope and colleagues [28] found that positive affect positively impacted general decision making. George and Zhou [29] revealed a relationship between positive affect and entrepreneurial creativity and ideation. Positive affect also positively predicts opportunity recognition [30,31]. Positive affect influences outcomes for business, as well. Baron and Tang [32] found that higher degrees of positive affect predicted sales growth and innovation. They also found that the benefits of positive affect influenced smaller entrepreneurial ventures more than larger ventures. Cardon and colleagues’ model of entrepreneurial passion emphasizes the role of high-activation positive affective states such as joy, energy, excitement, and enthusiasm. These states lead to key performance outcomes for the health of the nascent organization. Furthermore, an entrepreneur’s energy leads to persistence in the face of adversity [6].

The entrepreneur’s absorption in the activities of venture creation results in higher quality products and services. The entrepreneur’s ability to solve problems also creatively helps the organization carve its niche in the marketplace. The mechanism behind these outcomes can be explained through broaden and build theory [11]. Building on affect-as-resource theory and affect-as-information theory [18,33] broaden and build theory suggests that positive affect broadens thought-action repertoires and acts as a resource that helps one persist and learn from negative information [19]. Entrepreneurship entails risk and reward and is fraught with dangers and uncertainty. However, positive affect is not the only source of information for entrepreneurs; negative affect may also play a role. Brundin and Gustafsson [34] found that entrepreneurs’ likelihood to persist in a failing project increased with increased positive emotion (hope, self-confidence, and challenge in their study) and decreased with stronger feelings of embarrassment, frustration, and strain.

### 1.3. Negative Affect

Negative affect includes emotions such as anger, fear, shame, and nervousness [4]. Negative emotions exist independently of positive emotions and can, therefore, be experienced at the same time as positive emotions [35]. Affect-as-information theory suggests that negative affect informs a person that things are not going well [19].

Negative information represents a potential threat to survival, as it is attended to and processed more thoroughly than positive emotions [36]. Even though entrepreneurs experience fewer negative emotions than those who work for wages [9], successful management of negative emotions is a key factor in successful entrepreneurship [37]. Because entrepreneurs have more decisional autonomy than people who are employed by others, they can use more problem-focused coping mechanisms to overcome adversity [7].

Overall, a mix of positive and negative emotions may be optimal for entrepreneurship. Mixed and conflicting emotions are an important predictor of risk perception, which helps people avoid the common trap of overconfidence [38]. Baron [5] found that an excess of positive affect yielded diminishing returns, with a medium amount of positivity being optimal. While positive affect enhanced leaders’ abilities to recognize entrepreneurial opportunities, too much positive and negative affect both lowered their likelihood of acting on those opportunities [13].

### 1.4. Entrepreneurial Intention

Birthed by Bird’s [39] conception, EINTs shape the form, direction, and development of an organization. It links the entrepreneurs’ ideas and attitudes to their entrepreneurial behavior. Thompson [40] added that while conviction and planning are essential to EINT, actual venture creation is not. This is because entrepreneurial activities are rare and difficult to measure. For example, what constitutes “planning”? Is it the spark that ignites the thought or feeling that starting a business would be desirable or does a formal business plan need to be in place? Even though business start-up may not be the inevitable outcome, EINT is a strong predictor of behaviors [41] and is measured as a continuous versus dichotomous variable. This explains its use not only as a proxy for entrepreneurial behavior but also, as Thompson [40] suggests, as a construct that can stand on its own and be used as an independent and control variable.

The Theory of Planned Behavior (TPB) [41] is one of the most cited unidirectional models of intention. According to TPB, three factors, social norms, attitudes and perceived control, work in concert to influence the intention to act. Social norms refer to the perceived acceptance or aversion toward a specific behavior of those the target deems important in their immediate social environment. Attitudes are the target’s judgments and evaluations of the behavior. Lastly, perceived control is consistent with self-efficacy, the belief in one’s capability to perform specific tasks. Using the TPB framework to describe conscious intent, we define EINT as the desire and plan to start a business.

Affect-as-information theory [18] suggests that affect attunes people to the safety of conditions in their environment. Positive affect tells people all is well and they can relax, while negative affect leads people to search their environment for threats. Therefore, positive affect may tend to make people approach new situations more confidently, making them more likely to move forward with starting a business. This leads us to the hypothesis:

**Hypothesis** **1a** **(H1a):** *Higher positive affect is correlated with higher EINT.*

Further, negative affect may heighten people’s estimation of threats to successful entrepreneurship, making would-be entrepreneurs less willing to trust their vision, and therefore less likely to create new ventures. Therefore, we propose that:

**Hypothesis** **1b** **(H1b):** *Higher negative affect is correlated with lower EINT.*

### 1.5. Entrepreneurial Intention and Gender

An extant amount of research highlights interest in investigating the role of gender in entrepreneurial processes [42,43,44,45,46]. Even though research has resulted in some mixed findings [45,47] when comparing women and men’s EINT, copious research indicates that women report lower EINT compared to men [15,16,43,47]. The main reasons cited for differences between men and women’s EINT are (1) perceptions that the characteristics necessary for successful entrepreneurship are stereotypically male, (2) lack of training for women, (3) unfavorable economic and social environments for women, (4) lack of education for women, and (5) a lack of entrepreneurial self-efficacy among women [48,49]. Regardless of why this occurs, we have no reason to expect this study to be inconsistent with the abundance of past studies supporting a lower reported EINT of women compared to men. However, a better understanding of influential factors on EINT is an important step to closing the gap.

**Hypothesis** **2a** **(H2a):** *Women will report lower EINT than men.*

### 1.6. Affect and Gender

Social constructivist theories suggest that social norms and roles regulate emotions by signaling appropriate and valued responses [50]. Social roles held by men and women heighten sex differences in emotions and social behavior between men and women [51]. As suggested by Eagly and Wood [52], men and women are likely to possess sex-differentiated skills, beliefs, and subjective experiences that enhance the enactment of sex-typed social roles [52]. For example, women are socialized at an early age to be nurturing and develop verbal skills whereas men are encouraged to be aggressive and develop math skills. These socialization norms are reinforced through parents, media, and peers [53,54,55,56,57,58].

Consistent with social role predictions, women experience and display emotions congruent with expectations of their gender. Women report experiencing pleasant stimuli such as happiness more intensely than do men [59,60,61]. Additionally, women also suppress negative emotions and display positive emotions more than men [62]. Alexander and Wood’s [59] review of research highlights that women report more intense positive emotions than men, they more frequently express such emotions to others, and they respond more extremely to certain psychophysiological measures.

Thus, if women experience positive emotions more intensely, suppress negative emotions, and display positive emotions more than men, we suggest that they will confer the benefits of an approach mindset and process opportunities more readily than men. The benefits of positive affect, such as broadening one’s mind to possibilities, may open them to the idea of starting a business more easily than men. Therefore, we expect positive affect to enhance EINT. However, we expect this relationship to be greater for women than men because women’s socialization and past gender-role-related experiences are likely to instill subjective positive emotions and ways to respond to emotions that are congruent with their gender.

**Hypothesis** **2b** **(H2b):** *Positive affect will moderate the relationship between gender and EINT, such that it will be more influential on women’s EINT than men’s.*

Role congruity theory suggests that entrepreneurship would be challenging for women because the role of the entrepreneur is male-typed [43,63] and contrary to that of the stereotypical female gender role, whereas the male stereotype of entrepreneurship would confer a benefit for men. Numerous gender stereotypes surround women and are based on perceptions of what women’s roles are: from women’s views on education [64] to having nurturing personalities [65], which construct the way women approach career-related decisions. Examples of traditional gender roles would be that males are breadwinners and leaders of the family, whereas women are the homemakers and caretakers of the family. Consequently, acceptance of traditional gender roles may provide a boost for entrepreneurial men but a hindrance for women. Therefore, we hypothesize the following:

**Hypothesis** **3** **(H3):** *Acceptance of traditional gender roles moderates the relationship between gender and EINT such that stronger acceptance of traditional gender roles increases men’s EINT and decreases women’s EINT.*

**Figure 1:**
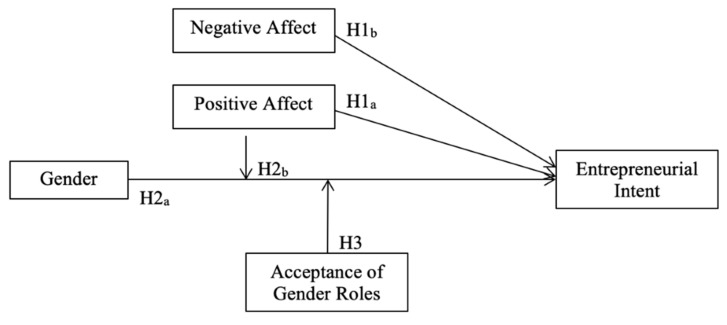
Model of positive and negative affect, gender, gender roles, and entrepreneurial intent.

Figure 1 shows the hypothesized research model of the relationships between gender, positive and negative affect, acceptance of gender roles, and EINT.

## 2. Materials and Methods

The data used for this paper were part of a larger study that utilized an internet-based survey and examined multiple constructs including EINT, acceptance of traditional gender roles, career choice, entrepreneurial industry interest, positive and negative affect, marketing strategies, and kidpreneur activities. The present study explored positive and negative affect, EINT, gender, and acceptance of traditional gender roles. The following is the full set of procedures used to administer the survey in the original study.

### 2.1. Participants

A convenience sampling method was used to recruit participants for the original study in 2019. Recruitment efforts included posts on social media such as Facebook and LinkedIn, referrals from friends, family, associates, as well as undergraduate and graduate students from four colleges in the Midwest, South, and Western parts of the United States. All students (*n =* 504) were offered a maximum of 2% extra-credit to take the survey.

Upon completion of data collection, the sample consisted of *n* = 928 subjects. Cases were examined for missing data and 76 cases were removed because of missing responses. The sample was narrowed to those responding as male or female, as only three participants identified as bigender. This left a usable sample size of *n* = 849 cases.

The ethnic diversity of the sample was homogeneous in that most participants (69.2%) identified as Caucasian. The ages of the participants ranged from 18 to 79 years with a mean of 26.2 years (*SD* = 10.86). Only about a quarter of the participants had earned an undergraduate or graduate degree (22.6%), 39% had completed some college, and about 21% had earned an Associate’s degree. Lastly, 446 participants had attempted to start at least one business or made at least one attempt to earn money in a self-employed way as an adult (M = 2.34, *SD* = 1.74, R*a = 1–10 or greater*).

### 2.2. Measures

Dispositional affect was measured using the 10-item International Positive and Negative Affect Scale (I-PANAS-SF; Thompson, 2007), an international shortened version of the PANAS [3]. The stem question, “Thinking about yourself and how you normally feel, to what extent do you generally feel:” was followed by five items for the positive affect scale and five items for the negative affect scale. The response set used a 5-point Likert scale ranging from one (*never*) to five (*always*). Two examples of items on the positive affect scale are *alert* and *attentive*. Two examples of items on the negative affect scale are *upset* and *hostile*. Both subscales achieved an acceptable level of reliability. Cronbach’s alpha was 0.80 for the positive affect subscale and 0.75 for the negative affect subscale.

EINT was measured using Thompson’s 10-item EINT measure [40], which has three items reverse-coded and four filler items and was assessed on a five-point Likert scale (1 = Does not describe me; 5 = Describes me extremely well). Two sample items include: *I intend to set up a company in the future*, and *I never search for business startup opportunities*. The Cronbach alpha was 0.82 for this measure.

Brown and Gladstone’s [66] short version of the gender role beliefs scale was used to assess the strength of participants’ acceptance of traditional gender role ideology. This measure has ten items. One of the ten items is reverse-coded. The measure used a one (*strongly disagree*) to 7 (*strongly agree*) Likert scale. Examples of items from this measure include: (1) *Women should be concerned with their duties of childbearing and house tending, rather than with the desires for professional and business careers*, and (2) *Swearing and obscenity are more repulsive in the speech of a woman than a man*. Cronbach alpha for this measure reached 0.75.

## 3. Results

### 3.1. Correlations

The analysis (see Table 1) showed several significant correlations among the independent, dependent, and two demographic variables. Positive and negative affect were negatively correlated with each other, *r* = −0.19, *p* < 0.01. EINT had a significant and positive correlation with positive affect *r* = 0.28, *p* < 0.01. And a significant negative correlation with negative affect *r* = −0.10, *p* < 0.01. This suggests that both higher levels of positive affect and lower levels of negative affect are associated with greater intention to start a business. Of note in the demographic variables, the number of business started was significantly and positively correlated with Age (*r* = 0.11, *p* < 0.01), Education (*r* = 0.09, *p* < 0.05), and EINT (*r* = 0.22, *p* < 0.01).

### 3.2. Hypothesis Testing

In all the following analyses, the number of businesses started as an adult was the only control variable. This was done because age, education, the number of businesses started were correlated and because research shows prior business ownership to be predictive of EINT [67,68]. As shown in [69], centering variables to mitigate potential threats of multicollinearity in unnecessary. However, we have done this to alleviate concerns. Furthermore, as can be seen in Table 1, the correlations between variables are fairly small. Therefore, the threat of multicollinearity is mild at best.

Hypothesis 1a stated that positive affect would lead to higher levels of EINT and Hypothesis 1b stated that negative affect would lead to lower levels of EINT. Hierarchical linear regression was conducted to test these hypotheses. EINT was entered as the dependent variable, positive and negative affect were entered as the independent variables, and the number of businesses was the control variable. The overall model was significant F(3824) = 36.69, *p* < 0.001. As can be seen in Table 2, the model explained twelve percent of the variance in EINT, R^2^ = 0.12, *p* < 0.001. Positive affect was a significant predictor of EINT, Unstandardized B = 0.34, SE B = 0.05, t(827) = 7.54, *p* < 0.001. However, negative affect did not predict EINT (*p* > 0.05). Therefore, we found support for Hypothesis 1a but not for Hypothesis 1b.

**Figure 2:**
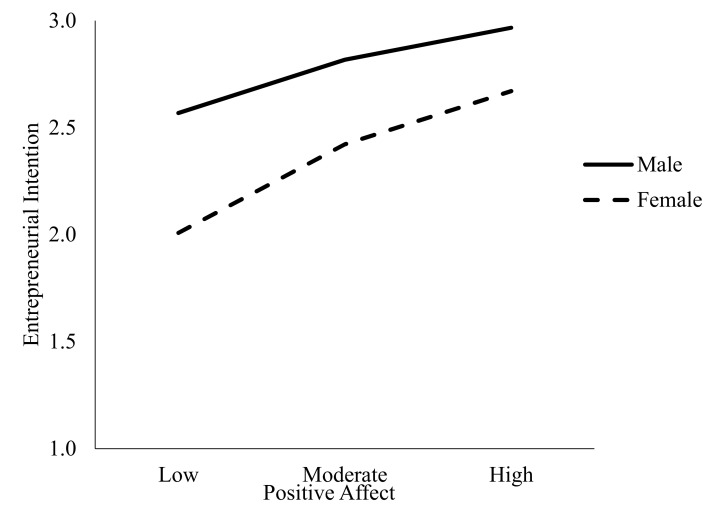
Interaction of positive affect and gender on entrepreneurial intention.

Hypothesis 2a stated that women will report lower EINT than men and Hypothesis 2b argued that positive affect will moderate the relationship between gender and EINT, such that it will be more influential on women’s EINT than men’s. Ordinary least squares regression was used to test these hypotheses by employing Hayes PROCESS macro-regression analysis (Model 1). A 5000-bootstrap sample was conducted to test this hypothesis [70]. Gender was coded zero for male and one for female. Controlling for the number of businesses, EINT was entered as the dependent variable and gender as the independent variable and positive affect as the moderator. The overall model was significant F(5, 822) = 32.31, *p* < 0.001, and explained sixteen percent of the variance R2 = 0.16 (See Table 3). Examination of the unstandardized coefficients revealed a significant main effect for the number of businesses started, B = 0.10, SE B = 0.02, t(827) = 5.51, *p* < 0.001. This result suggests that for each additional attempt at business ownership, EINT is predicted to rise 0.10 (one-tenth) of a unit. There was also a significant main effect for gender, B = −0.42, SE B = 0.07, t(827) = −6.48, *p* < 0.001. Lastly, the interaction was also significant B = 0.17, SE B = 0.09, t(827) = 1.94, *p* < 0.001. Based on the negative coefficients, the mean scores for EINT by gender (male mean = 2.79, *SD* = 0.95; female mean = 2.35, *SD* = 1.01) and the regression slopes which show a steeper slope for women than men, shown in Figure 2, show that women report lower levels EINT and that positive affect positively influences this relationship. Therefore, Hypothesis 2a and Hypothesis 2b were supported.

**Figure 3:**
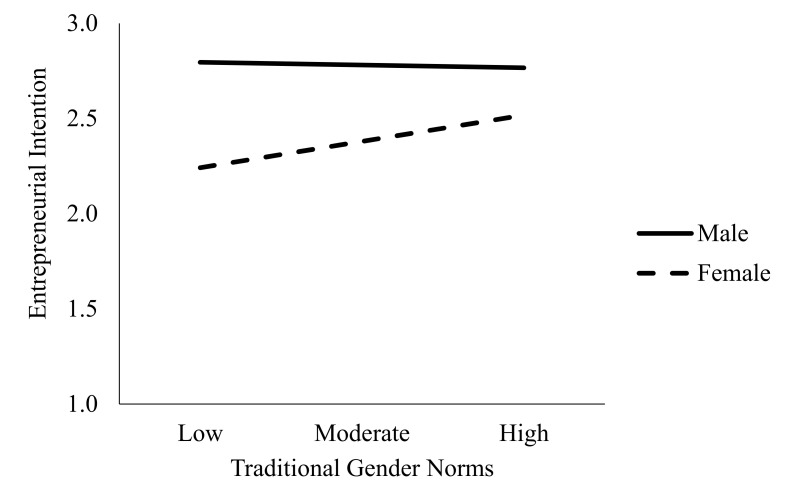
Interaction of Gender and Gender Norms on EINT.

Hypothesis 3 stated that stronger acceptance of traditional gender norms would moderate the relationship between gender and EINT, such that, as acceptance of traditional gender roles increases, men’s EINT also increases, and women’s EINT decreases. Ordinary least squares regression was used to test these hypotheses by employing Hayes PROCESS macro-regression analysis (Model 1). A 5000-bootstrap sample was conducted to test this hypothesis [70]. Gender was coded zero for male and one for female. Controlling for the number of businesses participants started as adults, EINT was entered as the dependent variable, gender as the independent variable, and acceptance of gender roles as the moderator. As can be seen in Table 4, the overall model was significant F(4826) = 21.96, *p* < 0.001 and explained 10 percent of the variance in EINT, R^2^ = 0.10, *p* < 0.001. Examination of the unstandardized coefficients revealed that there was a significant main effect for gender B = −0.40, SE B = 0.07, t(830) = −5.70, *p* < 0.001 and acceptance of gender roles B = 0.07, SE B = 0.03, t(830) = 1.99, *p* < 0.05. This result suggests that for each additional step in acceptance of traditional gender roles EINT is predicted to rise 0.07 (one-seventh) of a unit. A significant interaction was found between acceptance of traditional gender roles and gender, B = 0.15, SE B = 0.07, t(830) = 2.21, *p* = 0.017. The interaction explained an additional five percent of variance (*p* = 0.03). However, the interaction was in the opposite direction (see Figure 3) than hypothesized. For men, EINT decreased slightly as acceptance of traditional gender roles increased. The slope was steeper for women and was also in the opposite direction, as predicted. Women’s EINT increased as their support for traditional gender roles increased. Therefore, Hypothesis 3 was not supported.

## 4. Discussion

This study adds to the research about entrepreneurial affect by relating positive and negative affect to EINT. This approach considers positive and negative affect to be relatively independent dimensions [35]. Our results extend the few other studies examining affect and EINT, by demonstrating that higher positive affect is associated with higher EINT and that positive affect influences women’s EINT more than men’s.

Positive affect fuels resilience, which helps entrepreneurs to persevere in deciding if a problem is worth solving, and then solving it [71]. The ability to persevere and decide where to spend your personal and financial resources is a critical factor in entrepreneurial success. Using the broaden and build theory, the development of emotional and interpersonal relationships is consistent with long-term success in entrepreneurship. Congruent with affect-as-information theory, positive affect can act as a resource for perseverance through adversity, which is critical to entrepreneurial success.

However, our results did not show that higher levels of negative affect were related to lower EINT. The relationship of negative affect to EINT has not been well studied in previous work. When positing on the relationship, we thought that a higher negative affect may deter potential entrepreneurial motivation. However, in this study, higher negative affect was not related to lower EINT. The correlational analysis revealed a statistically small and negative relationship between the two constructs, however, enough variance was consumed by prior business experience during the regression analysis, for it to become not significant.

Opportunities to engage in entrepreneurship may counteract the effects of negative affect, by providing mastery experiences. Mastery experiences allow people to learn and improve and thereby increase confidence [72] and motivation. In this way, negative affect may support systematic thinking that enables more focused attention on problem solving and opportunity recognition [73,74,75]. As negative affect is an inevitable part of the journey of entrepreneurship, successfully coping with negative emotions is an important part of the successful entrepreneurship trajectory [7].

### 4.1. Implications for Theory

Our result showing that women’s EINT is lower than men’s EINT is consistent with previous work [15,16,43,47]. However, our novel finding that positive affect has more impact on the EINT of women than of men suggests that women can confer a benefit from stereotyping. Women are socialized to display more positive affect, therefore experiencing positive affect allows them more congruency with their gender role, making the road to entrepreneurship smoother.

As for the surprising result regarding the non-relationship between acceptance of gender roles and EINT, we recognize three possible explanations. First, women may report the belief that traditional gender roles should exist [76] yet consider entrepreneurship from a feminized perspective. For example, entrepreneurship can be used to supplement the household income. Based on the larger dataset from which these data were drawn [77], it was found that women tended to report the desire to start businesses in more feminized industries that are amenable to income replacement versus wealth generation.

Second, a shift in entrepreneurship may allow for easier entry and the ability for women to start a business and maintain a more traditional female gender-role. Technology has democratized Internet access, which enables entrepreneurs to tap into a larger audience of potential buyers than ever before and has made the location of the business almost irrelevant. Both factors allow women more flexibility to work around family duties. Perhaps this shift is changing entrepreneurship from a rarefied pursuit, only for the well-financed and well-connected, to one enabling women to connect with entrepreneurship in a way that is more congruent with stereotypical female gender roles.

Third, meta-analysis shows that gender roles and stereotypes are changing around perceptions of women’s roles [78]. As such, staying at home to take care of the family and home as women’s only responsibility may be fading from the female stereotype. Additionally, it may be that stereotypes about women in entrepreneurship are changing [43], or at least being cognitively neutralized [79]. The women in Gupta and colleagues’ study [79] showed that women, unlike their male counterparts, perceive entrepreneurs to have male and female characteristics. Imbuing the idea of entrepreneurship with female characteristics may allow women to reconcile cognitive dissonance between their gender-role and the entrepreneurial role.

### 4.2. Implications for Practice

These results are relevant for leadership and entrepreneurial training programs, especially those aimed at women. Building positive emotions over time can lead to more positive affect [2,3]. Focusing on providing positive experiences and producing higher positive affect within programs can help women increase their self-efficacy and increase the chances for successful entrepreneurship, thereby, increasing benefits to society such as job creation, new markets, and innovation.

For example, an examination of emotion in leadership training sessions for entrepreneurs [80] revealed that when entrepreneur participants spoke of their companies, they used language that was more emotive than rational, particularly if they were speaking about their fear of failure. Part of the emotional salience sprang from the fact that the entrepreneurs’ identity was entwined with their business. In the second day of training, the entrepreneurs engaged in difficult and potentially frustrating activities. During the debrief, the participants realized that they were being manipulated in order to understand their emotions and that, framed appropriately, the experienced emotions became an impactful and remembered part of the training [80].

Early entrepreneurial training which teaches the core tenets of business ownership (e.g., market research, strategy, or finance) can include stories of and learning from other successful women who are small business owners, entrepreneurs, or serial entrepreneurs. Some research shows that the development of women’s entrepreneurial self-efficacy can be contextual to size of business, industry of the business, and the level of female representation within an industry [81]. Understanding the lessons learned from successful launches by other women entrepreneurs will encourage positive affect through hope and inspiration. The increase in positive affect will increase self-efficacy via vicarious learning and social persuasion [11,72].

Furthermore, positive affect can lead to more openness to new ideas, in turn building resources to use in future problem-solving. Women entrepreneurs can use those resources in new and creative ways to implement feasible solutions and in opportunity recognition or venture creation [11,73]. Building positive affect during training is the start of what can flourish into positive outcomes for the would-be business, such as sales growth and innovation. This is an important factor because many women tend to migrate to industries with little growth that are not attractive candidates for financing [82].

Negative affect is not a barrier to future action, but rather information about challenges that need solving. Focusing attention on negative affect in training can include role-playing of difficult emotional conversations and discussions of venture failure [7]. In this way, would-be entrepreneurs are not blind-sided by the intensity of feelings that they may experience in creating their business.

In sum, opportunities to broaden thought repertoire can lead women to consider new ideas and ways of doing business and help dispel the myths about women entrepreneurs [83], such as not having the financial savvy or resources to start high-growth businesses, or that women do not have the right kinds of experiences.

### 4.3. Strengths, Limitations, and Implications for Future Research

The main strength of this research is that we add to the literature examining positive and negative affect, EINT, and gender which, to our knowledge, have never been studied simultaneously. Another strength can also be considered a limitation: the utilization of a homogeneous sample. Our sample was predominately students from state universities and professional adults across the United States. According to a study from the Ewing Marion Kauffman Foundation, most entrepreneurs find that a college education is important [84] and most entrepreneurs have studied in college, even if they have not received a degree. Therefore, our sample may be representative of would-be entrepreneurs. When researchers are limited to convenience samples, homogeneous samples convenience samples are a positive alternative because they can generalize to a subpopulation [69]. However, this strength is, of course, also a limitation, as we discuss.

A convenience sampling procedure was used to recruit participants and a large portion of the sample were students from state universities and professional adults across the United States. As with all samples of convenience, the benefits of ease of access to participants and relatively low cost for researchers on a shoe-string budget must be weighed against the disadvantages of lack of generalizability to the population as a whole, under- or over-representation of the population, and biased results based on participants’ willingness to take part in the research. As stated earlier, entrepreneurs consider education important and have engaged in some higher education [84]. Therefore, the sample for this study is reasonably generalizable to an American college-educated population and would-be entrepreneurs but not to the population as a whole.

Another limitation of the study, as with survey studies, is the potential for common methods bias. Common methods bias has been defined as “Response tendencies that raters apply across measures, similarities in item structure or wording that induce similar response, the proximity of items in an instrument and similarities in the medium, timing, or location” ([85], p. 476). Since positive and negative affect and EINT data were collected at the same time in this study, temporal separation was not available between the measurement of positive and negative affect and EINT variables.

Future research can provide more granularity on why women are less likely than men to engage in entrepreneurial activities. A longitudinal study to better assess EINT and positive and negative affect over time could help disambiguate how affect, emotion, and mood impact EINT.

## 5. Conclusions

The current economic environment in response to COVID-19 shows women’s unemployment to be 10.5%, while men are at 9.4% [67]. Although many men are stepping up to increased responsibilities in the home during lockdown, including childcare and schooling, women still bear the largest burden for these tasks [68]. This pandemic may change how women see entrepreneurship, create opportunity for online learning situations, as mentioned in the practical implications, and even provide more opportunity for digitally based businesses.

Entrepreneurs are necessary to both global and local economies in that the personal risks they take create new jobs, explore new markets, and contribute to innovation. By any measure, entrepreneurship is stressful. More positive affect can help entrepreneurs build resilience, positively adapt to stressful situations, and tolerate ambiguity. Potential entrepreneurs, and particularly women, will have more tools to use in uncertain times. By casting more light on the ways that affect impacts entrepreneurship, we can help create stronger companies, more meaningful work, and reap the full benefits of entrepreneurship.

## Figures and Tables

**Table 1:** Correlations of independent, dependent and demographic variables.

	Measure	Mean	*SD*	1	2	3	4	5	6	7	8
1.	Age	26.21	10.86	-							
2.	Businesses	1.23	1.72	0.11 **	-						
3.	Education	4.03	1.63	0.48 **	0.09 *	-					
4.	Gender	0.54	0.50	−0.03	−0.09 **	−0.05	-				
5.	NA	2.01	0.57	−0.09 *	−0.04	−0.09 *	0.08 *	-			
6.	PA	3.61	0.76	0.00	0.10 **	0.11 **	−0.02	−0.19 **	-		
7.	STGR	3.04	1.0	0.03	0.06	−0.12 **	−0.24 **	−0.04	0.78	-	
8.	EINT	2.55	1.01	−0.08 *	0.22 **	0.065	−0.22 **	−0.10 **	0.28 **	0.13 **	-

Note: Businesses = number of business started or attempted to earn money through self-employment as an adult; Education codes 1 = Did not finish high school, 2 = Earned high school diploma, 3 = Completed some college, no degree, 4 = Completed a trade school certified, 5 = Earned a 2-year college degree, 6 = Earned a 4-year college degree, 7 = Earned a master’s degree, 8 = Earned a professional degree (e.g., JD or MD), 9 = Earned a PhD; Gender coded 0 = male, 1 = female; NA = Negative affect; PA = Positive affect; STGR = Support for traditional gender roles; EINT = Entrepreneurial intention. * *p* < 0.05, ** *p* < 0.01.

**Table 2:** Results of regression predicting entrepreneurial intention (EINT) based on positive and negative affect.

Predictor Variables	ΔR^2^	B	SE B
Model 1			
Businesses started as adult	0.05 ***	2.34 ***	0.04
Model 2	0.07 ***		
Businesses started as adult		0.12 ***	0.02
Positive affect		0.34 ***	0.05
Negative affect		−0.52	0.76
Cumulative R2	0.12 ***		
Adjusted R2	0.12 ***		

Note: Business started as adult response range 1, 2, 3, 4, 5, 6, 7, 8, 9, 10+. *** *p* < 0.001.

**Table 3:** Results of regression predicting EINT based on gender and positive affect.

Predictor Variables	ΔR^2^	B	SE B
Businesses started as adult		0.10	0.02 **
Gender		−0.42 **	0.07 **
Positive affect		0.34 **	0.04 **
Interaction gender by Positive affect	0.01 *	0.17 *	0.09
Cumulative R2	0.16 **		

Note: Business started as adult response range 1, 2, 3, 4, 5, 6, 7, 8, 9, 10+. * *p* < 0.05, ** *p* < 0.001.

**Table 4:** Results of regression predicting EINT based on gender and support of gender roles.

Predictor Variables	ΔR^2^	B	SE B
Businesses started as adult		0.11	0.02 **
Gender		−0.39 ***	0.07 **
STGR		0.07 ***	0.03 *
Interaction gender by STGR	0.01 *	0.15 *	0.07 *
Cumulative R2	0.10 ***		

Note: Business started as adult response range 1, 2, 3, 4, 5, 6, 7, 8, 9, 10 +; STGR = Support for traditional gender roles. * *p* < 0.05, ** *p* < 0.01, *** *p* < 0.001.
